# Linguistic and Material Ways of Communicating with Cows—The Dung Pusher as a Semiotic Resource

**DOI:** 10.3390/ani16020201

**Published:** 2026-01-09

**Authors:** Anni Jääskeläinen

**Affiliations:** 1 Department of Finnish, Finno-Ugrian and Scandinavian Studies, University of Helsinki, Unioninkatu 40, 00170 Helsinki, Finland; anni.m.jaaskelainen@helsinki.fi; 2 Helsinki Institute of Sustainability Sciences HELSUS, University of Helsinki, 00100 Helsinki, Finland; 3 Finnish Literature Society, Hallituskatu 1, P.O. Box 259, 00171 Helsinki, Finland

**Keywords:** linguistic animal studies, biosemiotics, human–animal studies, human–animal communication, multi-species pragmatics, gesture studies, multimodal conversation analysis, Umwelt

## Abstract

This study examines how farm workers working with cattle talk to and interact with these non-human animals. This study presents linguistic animal studies and multi-species pragmatics, and it is based on fieldwork, interviews, and video recordings from several types of Finnish dairy farms. This study concentrates especially on one facet of human–cattle interaction: how humans use dung pushers and other sticks when communicating with cows. Thus, it draws on the materiality of language. It is shown how objects, bodies, and spaces, as well as words and linguistic constructions, are meaningful in human–animal interaction. Videoed recordings are analysed with multimodal conversation analysis. It is shown how dung pushers and snow stakes are used when steering cows, making them stand up, and pointing at things. It is then shown how these objects become ‘meaning-carriers’ for humans and for cows. For example, the dung pusher acquires four different meaning qualities for the human participants in the cattle barns: floor-cleaner quality, shepherd’s-crook quality, pointer quality, and weapon quality. The study examines how the cows’ and humans’ Umwelts, the subjective meaning universes of these species and their constituent individuals, influence interaction on farms and how and why the dung pusher becomes a semiotic resource.

## Simple Summary

This study explores how Finnish dairy farm workers communicate with cattle using different modalities of communication: spoken language, touch, gestures, and material objects. The study is based on fieldwork, interviews, and video recordings from dairy farms. The study shows how two types of sticks, dung pushers and snow stakes, are used in a cattle barn when communicating with cows. The way these sticks are used turns them into “meaning-carriers”, which means that they become more than just tools for cleaning floors or marking roadsides. The way dung pushers and snow stakes become “meaning-carriers” depends on the farm’s setup and the cows’ living conditions. The article discusses the biosemiotic concept of “Umwelt,” a unique world of experiences and meanings for each species, and emphasizes the importance of trying to understand animals’ perspectives. This research highlights that cows and humans share a living space full of meaning, and that the space and its material objects constitute a world of multi-dimensional meanings for both humans and cows.

## 1. Introduction

This article studies how dairy cows are talked to in certain modern-day Finnish dairy farms, as part of the everyday life of these production animals and their caretakers. During the day, there are many occasions during which farm workers talk to and with cattle, and they talk to cattle in many different ways. However, for this study I have chosen just one example of these talking instances: the episodes found in my research data where farm workers use dung pushers or other sticks, such as snow stakes, when communicating with cows. I study the linguistic as well as material affordances (see [1], pp. 626–627) that people make use of in these communications. Thus, this approach takes into consideration the materiality of language (see [2]) and the linguistic, indexical, and other meanings available to humans and cows in these situations. I call cows *animals* in this article. It is a shorthand for *non-human animals*. I am aware that humans, too, are animals and that we share many cognitive and bodily faculties with our non-human co-habitants of this world. By choosing this topic for my research, I am not committed to, or agreeing with, the way that farm animals are farmed or treated when farmed. I wish to study the reality and conditions of these animals as they are, and I also wish to understand the very complex situations and trajectories that have led people and animals to this reality, and what it is like for them.

### 1.1. Academic Background and Orientation

This article represents linguistic animal studies [3,4], and I place myself as a researcher of human–animal communication (see, for example, [5,6,7]). These fields are not experimental animal sciences but a part of the more general field of human–animal studies that often makes use of qualitative research. Although I study language and grammar, I acknowledge the fact that language presents just one modality through which meaning is conveyed in the multi-species situations I study. I am inspired by the research of biosemioticians and zoosemioticians, who see the world as full of meaning, different kinds of meanings for different creatures in their respective meaning universes (see [8]). These subjective universes, *Umwelts* [9], present many intricate layers of semiosis. Eduardo Kohn [10] (p. 55) writes, “The semiosis of life is iconic and indexical. Symbolic reference, that which makes humans unique, is an emergent dynamic that is nested within this broader semiosis of life from which it stems and on which it depends”. Indeed, humans are animals too, and, in addition to symbolic meaning systems, such as the language we use, we have access to other layers of meaning, some of which we share with cattle, thanks to our mammalian bodies and senses, even if our Umwelts are not nearly the same. In this article I also examine what meanings—iconic, indexical, and symbolic—we find in the cattle barn. Thus, the use of *meaning* in this study is not in its typical use in a linguistic study, where it would refer to semantic and (sometimes) pragmatic aspects of meaning (see [11]), but is more general and embodied.

### 1.2. Why Study Talking to Cows

I chose the use of sticks in communication as the subject of this study because the use of sticks shows an interesting aspect of the materiality of language (see Section 1.4). However, in my research data, cows are, for example, called, they are steered and directed, they are told what to do and what not to do, they are chatted with, and—quite often—stroked and baby-talked to. They are also used as a resource when talking to other people (see [12,13]) and they take part in “ventriloquizing”, which means that a human interlocutor pretends to talk as the cow, when aiming the message to a human nearby [12]. In the older written data, there are also accounts of singing to cows and sharing one’s own worries with them, being comforted by cows. (See [14], pp. 239–240.) Therefore, the examples of communicating with cows with dung pushers and other sticks in this study only show a small context of human–cattle communication and linguistic activity.

I study how stockpersons talk to cows in order to know how animals are talked to and what kind of language we use when talking to animals, which tells us something about human–animal relationships and language. As a rule, linguistic studies are not performed for any ulterior motive; the only motivation needed is to understand language and the people using language better. That being said, research shows that there are certain benefits in talking to cows.

Early on, it was realized that a good human–animal relationship (HAR) was a benefit, as it helped to yield larger milk quantities [15,16]. Studies have shown that talking to cows and calling them by their name is one indicator of a better HAR, which correlates with better welfare of the animals, as well as a larger milk yield [17,18]. Intuitively, many producers seem to equate talking to cows with good care and the well-being of cows [19,20]. Most studies on animal welfare agree that the stockperson is perhaps the most important environmental factor for cows and crucial to their well-being, e.g., [21]. In addition, when reading cow-care manuals written for stockpersons, gentle handling and speech are often mentioned as examples of good care, like in this passage: “Good interaction [with cows] includes calm and consistent handling, talking to animals with a friendly voice, and everything that creates a calm atmosphere” [22], p. 27. Accounts of cow mistreatment report shouting at cows and calling them names [23], pp. 78–79. Animals are talked to in either case—when they are respected and when they are mistreated. It is more of a rule that people taking care of cows talk to them, at least on certain occasions, than that they would not talk to cows. In my research, I attempt to find out how cows are talked to.

### 1.3. Tools in This Study: Dung Pusher and Snow Stake

**Figure 1:**
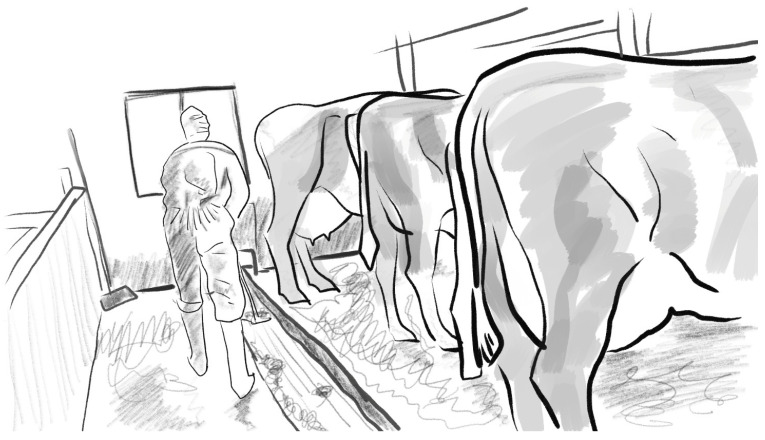
Using a dung pusher, Farm 2 of the 5 farms studied in the present investigation. Another dung pusher stands in the corner.

A dung pusher (*lantakola*, also called a *paskakola*, ‘shit pusher’) or a manure scraper is a tool meant for pushing dung into manure gutters and for arranging the cows’ bedding in cubicles. It is composed of a long shaft of wood (sometimes aluminium or fibreglass) and a flat edge, made of plastic or rubber, the size of which differs and is chosen depending on the size of the manure gutters. Farm workers are very familiar with this tool, and they often have one waiting in several places around the cattle barn. See Figure 1.

A snow stake (*aurauskeppi* or *aurausviitta*), on the other hand, is a relatively light, bendy, plastic stick, usually orange in colour, decorated with reflectors. Snow stakes are used when marking roads and paths during wintertime, so that the snowplough’s driver sees where the sides of the road are. Thus, this tool has, basically, nothing to do with cattle farming, except that the farmers probably have them anyway.

Both tools are used when communicating with cows: the dung pusher probably because it is there, and the snow stakes in a more planned way.

### 1.4. Materiality of Language

In the present study, the use of dung pushers and snow stakes is one example of the *materiality* of language [2,24]. Language is anchored in the material surroundings of its use in many ways. In Conversation Analysis (CA) the concept of the materiality of language has been used to explain the fact that objects and artifacts are used in many ways when accomplishing actions and activities [24]. In CA, as well as in sociology and sociolinguistics, there has been an “embodied turn” in the research of interaction [25]. In these fields, new concepts are being used in order to consider differing material elements in the study of interaction. These concepts include semiotic assemblages [26] and semiotic repertoires [27]. Interactional actions and activities take place in factual or virtual environments, and these surroundings are manipulated in many ways as part of the interaction [2,24]. The bodies of participants, their positions, postures, and gazes, are also a part of the context, unfolding in time. Thus, bodies of participants are among the semiotic resources, or “semiotic fields”, that participants have at their disposal [28]. In the cattle barn, the workers use their bodies and other elements in many ways when communicating with cows, and they orient themselves to the bodies of cows, reacting to their positions, gaze, and movements, for example.

A significant aspect of the materiality of language that surfaces when examining communications with the cattle is the *touch*. The cattle are touched, pushed, steered with hands, tapped, wiped down, stroked, scratched, and patted, and have their ears pulled gently, for example. (See [29], p. 219; see also [30].) Most of the farm workers I followed seemed to have an instinctual way of communicating with cattle with touches, as well as with words, and they touched the cows every now and then. Clearly, this was one of their attempts to convey their intentions and mood to cows. Touches also became indexes and routines in certain activities, such as pipeline milking (see [31]). Yet another facet of the materiality of language is the use of gestures and pointing (e.g., [32,33]), which, too, will be dealt with in this study.

### 1.5. Researcher’s Body in Intra-Action

Visiting the farms in my study was a thoroughly bodily experience. It was often quite cold inside the cattle barns. I could smell the cows, their fodder, their dung. I could hear the machinery: the loud, constant, and disconcerting thumping of milking robots, the sound of the automatic dung plough and other machinery. I could hear the insistent mooing of a new mother cow deprived of her calf, the mooing of other cows and calves, the sound of cows urinating on the floor, and the radio. I could see dung on the floor and on the walls. Birds were calling, flying outside and inside. I could feel the cold and, sometimes, the cows licking my hands and pulling on my clothes. I could see their expressive ears and eyes, their bony sides and protruding shoulders, and their large udders and difficulties standing up. But, of course, the sounds and sights were different in different farms, as the machinery, the premises, and the ways of keeping the cows were different. I realized that my body became one of my research instruments, as I started to sense the semiotic layers that were available for the cows and for us humans as bodily creatures sharing the same space. The cattle barn was full of meaning, but not always symbolic meaning. I also became fond of the cows and found their large, warm bodies comforting, and I enjoyed spending time with them.

The cows and the material environment thus affected me as an instrument and thus affected my studies. I must have affected the other participants, too, with my presence, questions, smell, and voice. Karen Barad and other post-humanistic thinkers call these kinds of entanglements intra-actions [34] (see, e.g., [35], pp. 314–318; [36], p. 4). Barad [34], p. 128, writes, “I introduce the term ‘intra-action’ in recognition of their ontological inseparability, in contrast to the usual ‘interaction,’ which relies on a metaphysics of individualism (in particular, the prior existence of separately determinate entities). A *phenomenon is a specific intra-action of an ‘object’ and the ‘measuring agencies’*; the object and the measuring agencies emerge from, rather than precede, the intra-action that produces them”. Embracing Barad’s notion of intra-action, this research also falls within the traditions of post-humanism and new materialisms. (See [37], p. 1.) I will now describe the methods and materials of this study.

## 2. Materials and Methods

This study is the result of ethnographic and autoethnographic fieldwork as well as linguistic analysis. The data for this study consists of my own field recordings, notes, and interviews on 5 different Finnish dairy farms (with 8.5 h of video-recorded footage and c. 4 h of recorded interviews), as well as the reality television show Lomittajat (2024), ‘Farm relief workers’, in its entirety (3.5 h). This television show follows the work of four farm relief workers on their workdays on 11 Finnish dairy farms. All participants have given their written consent to participate. All names in this and other data have been anonymized, and I will not give the exact locations of farms, as agreed in the informed consent form signed by informants. The purpose of the study and principles regarding voluntary participation, pseudonymization, and confidentiality were explained to the subjects prior to the study. Because of anonymization, photos or footage of the participants are not shown, and pictures are shown as drawings, as was also explained to participants in the informed consent form. I have obtained permissions to use Lomittajat (2024) as study material from the broadcasting company and the individual relief workers.

This examination includes different kinds of farms: small, medium-sized, and large; organic and non-organic; tie stall and free stall. I was able to see cows’ season inside in the late-autumn, and their season outside in the summer. All these different conditions had an influence on the practises of talking to cows and how the object that I examine in this article, the dung pusher, was used. For the purposes of this study, I call the farms in my study Farms 1, 2, 3, 4, and 5. All these farms are situated in Southern and central Finland. In Finland, dairy farms are, although growing in size, still relatively small compared to the rest of Europe and especially the rest of the world. In 2024, the average number of cows on a Finnish dairy farm was 55 [38].

Farm 1 is a free-stall cattle farm that farms organically, being the only organic farm in this sample. It is relatively large with c.100 milking cows. (In addition to milking cows, all these farms have calves, heifers, and dry cows that are not counted in as milking cows; most of these non-milking cattle are usually kept in different premises and on different pastures.) On this farm, the cows are able to go outside freely during winter and summer and are milked by robots, accessible to the cows at all times. Thus, the cows can move about relatively freely. The stockperson I followed on Farm 1 had c.20 years of experience in taking care of cattle, and they had been at this farm for 2 years. The owner of this farm (whom I also interviewed) had decades of experience in dairy farming. (The expertise of the stockpersons as such is not relevant in my research frame. I am not trying to decide if the stockpersons are experienced or not, or how ‘well’ they talk to cows.)

Farm 2 is a small dairy farm with a tie-stall cattle barn. This farm has c.15 milking cows, who are milked with pipeline milking. I visited this farm during the winter season. At that time the cows were inside and attached to their individual stalls with little space to move. The barn in Farm 2 was quite old and the most old-fashioned in my sample, with narrow spaces and wooden structures instead of metallic tie-stall loops. The stockperson followed in Farm 2 was a relief worker with 30 years of experience. The owner, interviewed for the study, had been with cattle all their life.

Farm 3 is a dairy farm with a free-stall cattle barn. This farm has c.600 milking cows, milked with a milking parlour three times a day and moved to the parlour in groups of 50 cows. The cows can move but spend their time inside the cattle barn. Relative to the Finnish scale of dairy farms, this farm is very large, one of the few of this size. Farm 3 was a relatively newly established farm with modern solutions. Its three co-owners each had a long experience in dairy farming. I did not visit farm 3 personally. I interviewed its owner remotely, and they sent me footage filmed by them according to my wishes.

Farm 4 is a tie-stall cattle barn farm with c.50 milking cows, which makes it quite big for a tie-stall barn farm [39]. The milking method is pipeline milking. I visited this farm during the summer season, which meant that the cows were mostly outside in a pasture, and they walked twice a day to the barn to be milked. The two stockpersons on Farm 4 that I followed had c.20 and 40 years of experience, respectively. One of them was the owner of this inherited family farm, and the other one was their relative.

Farm 5 is a meat and dairy farm with c.30 cows (in total). Keeping this farm is not the main occupation of the owner. I visited this farm in the summertime, after interviewing the owner. I did not see milking or the use of dung pushers on this farm, but the owner explained to me how they used sticks, so this farm is included in this study. The owner of Farm 5 had almost 60 years of experience in cattle farming, and their stockperson had c.10 years of experience.

Lomittajat: The television show Lomittajat, ‘Farm relief workers’, was shot on 11 different farms, and it presents differing ways of housing and milking cows. Both free-stall and tie-stall barns can be seen, as well as pipeline, robot, and parlour milking. In this study it will be referred to as Relief workers. The relief workers had differing amounts of experience, ranging between c.3 and 30 years.

This study is an example of qualitative research, using methods of qualitative data-based language analysis and (multimodal) CA. (See, for example, [40,41].) During the analysis phase, the field-notes and the footage were thoroughly examined, and specific episodes in the data were chosen for an even closer analysis. By an episode, in this specific study, I mean one communicative event in my footage in which a farm worker is trying to get a cow to do something by moving a stick, often while talking to the cow. In an episode of this kind, the stick does not necessarily touch the cow, but the movement is intentional and directed towards the cow. One episode might thus contain just one movement of the stick in the direction of the cow, or several touches to the cow made with the stick, as well as linguistic material. In addition, there are dozens of examples where a farmworker just holds a dung pusher or another stick in their hand, but these are not counted as episodes.

In this material, there were 29 episodes, in which sticks are used communicatively. The sticks used included dung pushers and snow stakes, white sticks the size of snow stakes, on one occasion a broomstick, and on one occasion an unidentified plastic stick. Out of these 29 episodes, I then chose the examples presented in this study. Examples 1 and 2 were annotated with the help of Elan 7.0 and then transcribed meticulously, using the conventions of multimodal transcription [42,43], modified for the purposes of this study; a careful transcription and its interpretation are crucial in showing the reader that they could come to the same conclusion when seeing the footage. This forms a part of the objectivity of conversational analysis. (See [44].) Reading these examples is difficult, especially for an untrained reader, and I advise the reader to see the Abbreviations section at the end of this paper. Other examples (4–7) were transcribed more simplistically to enable easier reading. The Leipzig glossing rules were used in morpheme-by-morpheme glossing of language examples. (https://www.eva.mpg.de/lingua/resources/glossing-rules.php, accessed on 1 September 2025). Pictures were drawn from actual footage with the help of Krita 5.2.2.

## 3. Umwelts and Meaning-Carriers—Four Different Types of Living Situations in My Data

The living conditions of animals and the working conditions of workers, their material surroundings, are of utmost importance when studying how and why cows are talked to, or, more generally, which kinds of linguistic and semiotic activities they partake in. These material surroundings include spaces, premises, machinery, tools, props, food, water, and even the season (see also [45]; the studies of Leonie Cornips (e.g., [45,46,47]) have very much inspired my work).

**Figure 2:**
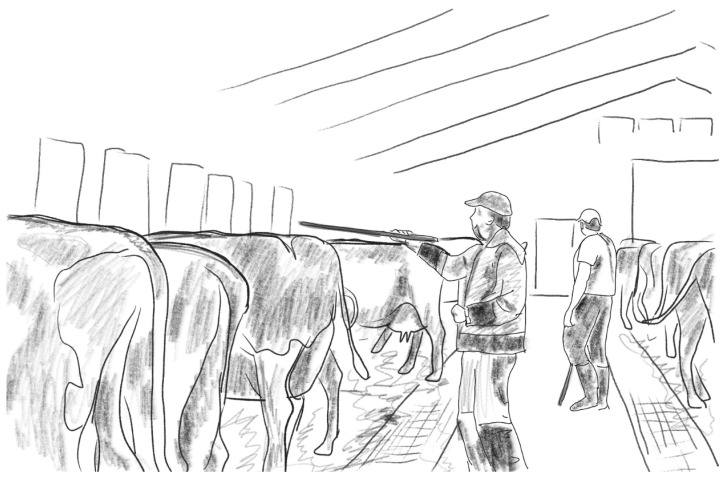
Herding cows with sticks, Farm 4.

These contextual factors could be simplified to four different conditions, as seen in the Table 1 below:

These four different ways of keeping the cows meant that the dung pusher, too, was used in a different way. On Farm 1, farm workers would walk most of the time with the dung pusher in their hand. The structure of the floor and the way their large cattle moved inside their barn meant that there was a lot of dung to be cleaned. On Farm 2, on the other hand, the cows were attached to their stalls, and the narrow manure gutters were just behind the stalls (see Figure 1). The floor cleaning process was thus quite different, as was the pipeline milking process. On Farm 3, there would be quite a lot of dung to be cleaned, but because the milking process was different, the dung pusher also served another kind of function, as I will soon explain: as a shepherd’s crook when moving the cattle into milking premises. Finally, on Farm 4, it was summertime and the cows were outside. As I was there, the workers used the dung pusher very little, because the cows mostly defecated outside. However, these cows needed to be herded to their cubicles, and the workers used snow stakes for this task. See Figure 2.

Jakob von Uexküll, who is considered one of the first biosemioticians, describes the *Umwelts*, the subjective meaning universes of living creatures [9]. (Von Uexküll’s seminal study [9] first appeared in 1940, in German, as *Bedeutunglehre*. Its English version was then published in the journal *Semiotica*, translated by Barry Stone and Herbert Weiner, in 1982. T. von Uexküll [48] emphasizes how demanding that translation task was.) An Umwelt is inherently a semiotic “place”: it consists of the things that bear meaning to a subject. Because different things are meaningful to different species and the senses of species differ significantly, even the same physical places become different for different species inhabiting them. Thus, the cattle barn does not present the same meaning universe for different creatures in it, for cows, for humans, for spiders, for swallows, for shepherd dogs, and so forth. Von Uexküll [9] calls the significant things that define one’s Umwelt *meaning-carriers*. Meaning-carriers are different for representatives of different species and, to some extent, for different individuals. We have different senses, attuned to be receptive to things meaningful to us, and many other things we simply ignore or have not the faintest notion of. One of von Uexküll’s [9] (pp. 29–31) examples is a flower: in the Umwelt of a human, it is for picking and has aesthetic value; in the Umwelt of an ant, it is a path; in the Umwelt of a cow, food; and in the Umwelt of a cicada larva, building material for a nest. Kohn’s [10] approach is similar to that of von Uexküll’s, although Kohn [10] mainly follows Peircian semiotics (which von Uexküll did not know [48]). Kohn [10] also realizes that the living spaces of humans and other animals are full of meaning.

Cattle (*Bos taurus*) are a domesticated species, living in confined premises built by humans, and are especially bred to produce as much milk or meat as possible, which has changed their bodies significantly. (See [49,50,51].) Cows’ Umwelts are thus partly different from the Umwelts of their predecessor, the auroch (*Bos primigenius*). Aurochs probably grazed in small herds in the forests, lakesides, and lowlands. Their senses were similar to those of the modern cows, but they did not live in industrial hall-like houses with slatted floors, manure gutters, and artificial light. They were not attached to wooden or metal structures, either too close to other members of their species or unable to touch them, or attached to machinery for milking. They ate what they found when grazing, not what the farmer gave them. Aurochs were not artificially inseminated and were able to live with their calves and nurse them as they liked, to name but a few differences. A domestic cow in a free-stall cattle barn might never see the light of day. According to Finnish law, cows kept in tie-stall cattle barns must be allowed to graze free, or at least be able to walk free, for 90 days per year; usually this means outside, but the law does not specifically state “outside” (*The Governments degree on cattle welfare*: https://www.finlex.fi/fi/lainsaadanto/2010/592, accessed on 1 September 2025). These 90 days only apply to cows kept in tie-stall cattle barns. Cows kept in free-stall cattle barns are “free” in the sense that, according to this law, there is no further requirement to let them go outside. Thus, cows in large free-stall cattle barns might never go outside. Although domestication has changed cows (e.g., [52]), the senses and instincts of the aurochs and the domestic cattle are much the same. However, they do not come across the same things in their lives; therefore, some of their meaning-carriers develop differently. An Umwelt is fundamentally the product of meaning-giving processes of a subject, in this case, an individual cow living in a barn.

The Umwelt of a domestic cow is very much dependent on its living conditions, of which the workers, the barn, and the milking method are the most significant (see [45,53]). Domestic cows soon come across many meaning-carriers that also become routines in their lives ([9], and Section 5). Some of them are, to us, linguistic signs, such as the volitive interjections and other directives used when calling and steering cows, but to cows they are probably indexical meaning-carriers (see [3,4]). These include, in my present data, the interjection *tsup tsup tsup tsup tsup* when hurrying a cow, and the interjection *tse* when calling them, as well as other directives. The owner of Farm 5 told me that they always teach cows certain directives. These included *nosta* (raise.imp.2sg), *käänny* (turn.imp.2sg), *tule* (come.imp.2sg), and *ei* (no), delivered with appropriate sound quality. The directives *nosta* and *turn* were used with bodily contact to the cows, a slight touch on the leg or side. This material part of the directives was a part of their form, at least in the beginning. In cognitive and constructional linguistics, it is agreed that a linguistic sign (a construction) is a conventional, symbolic assembly of form and meaning/function [54], (p. 3); [55], (pp. 15–21); [11,56], (pp. 14–16). In the beginning, the gesture of touching the cow was a part of the sign’s form, the meaning of which was [TURN IMPERATIVE]. When the cow learned the meaning of the verbal sign, the bodily part of the form was dropped, and the phonetic form alone supported the meaning. In the opinion of this informant, the cows soon learned these commands.

**Figure 3:**
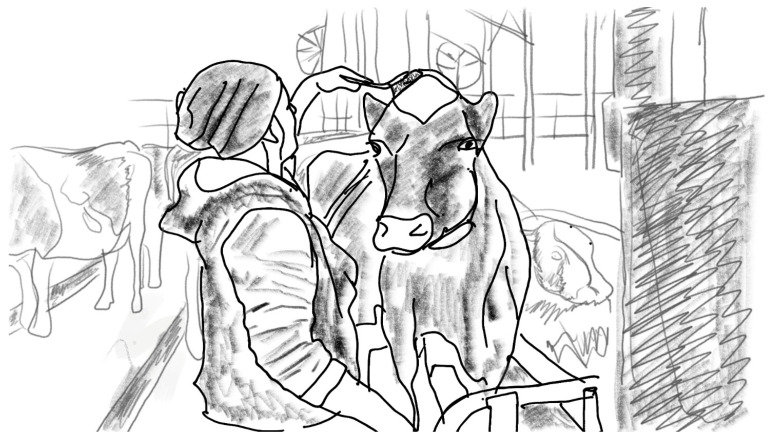
Cow having her hair done.

Not all cows’ meaning-carriers are linguistic. A meaning-carrier could be the farm worker’s bucket, which means that they will receive food (as on Farm 5), or the truck loader, which might mean the same thing (as it did on Farm 1). On Farm 3, one significant meaning-carrier was the gate that was separating the grazing hall from the corridor opening; the opening of the gate meant that the cows must move to another space to be milked. On Farm 1, one cow had a very specific meaning-carrier: when the farm worker took out their dish brush in order to clean the drinking sinks, this cow came straight up, because she wanted to be brushed, “to have her hair done”, as the farm worker said. (See Figure 3.) The cow had learned about this meaning-carrier before this specific worker had started there, and the cow had actually taught the worker about it. This recurring action was meaningful for both of these individuals.

Because of the differing living conditions of cattle farms, the dung pusher also became a different meaning-carrier for people and for cows, as I will describe in more detail in the following sections.

## 4. Examples of Dung Pusher Use

In this section I analyse more closely 2 of the 29 episodes of my data in which humans use sticks and language in performing an action involving cows, and I also present 2 examples in which a dung pusher or a snow stake is used as a pointer; in these examples a person does not communicate with a cow but with another person. In Section 5, it is explained which meanings the dung pusher and snow stake thus acquire in the farm context.

### 4.1. Directives and Commands in Steering Cows

In the 29 episodes of using sticks in a communicative way in my data, the human participant is steering a cow towards the direction they have chosen (for example, the milking parlour, a milking robot, the barn, or a specific cubicle inside the barn), or they are trying to make a cow move or get up. If we look at the type of linguistic activity in these 29 episodes, they contain directives of different kinds [57], (§ 1645). Directives represent commands as speech acts [58], (p. 2).

Specific directives used in these episodes are morphologically different. There are imperatives, which are the prototypical commands [58]: *Mee pää pöytään*, go.imp.2sg head.nom table.ill, ‘Go head to table’; *Nouse nyt*, rise.imp.2sg. now, ‘Get up now’; *Nouse*, rise.imp.2sg, ‘Get up’; and *Mene-hän Melissa*, go.imp.2sg-ptcl Melissa, ‘Go on Melissa’. (All names in my data have been pseudonymized, including cows’ names; however, as the initial in cow names is meaningful, as it indicates the year the cow was born, I have kept the initial the same.) There are also second-person singular indicatives, which are used as strong commands (*Et lähe*, no.ind.2sg leave, ‘You don’t leave’), and passive forms, also typically used in Finnish commands (*Käännytää sinne,* turn.pass there, ‘Let’s turn that way’; *Mennää-s* go.pass-ptcl, ‘Let’s go’), as well as particles, such as *No ni*, ‘well’, ‘there’; *Heippa*, ‘hey’; *Ylös* ‘up’.

The linguistic part in these actions is often quite minimal. In many cases it consists of just one directive word, said once or repeated, as in example 1 below, *juu juu* ‘yes yes’ and *no ni* ‘well’, ‘that’s it’. In many cases, the action is performed only bodily, using the ‘shepherd’s-crook quality’ of the dung pusher or snow stake (see Section 5). For example, on Farm 4, there were eight episodes of steering cows with snow stakes, and only one of them contained any words, the command being *Tänne päin*, ‘this way’.

Perhaps we could state that the action of steering cows is more bodily than it is linguistic. It comprises movements and touches and noises made with sticks, such as clattering against metal, and only sometimes words. In addition, the project of moving the cows or trying to get them up is the human participant’s project, and the cow might have—and often has—her own project. In example 1 below, the project of the cow is to rest in her bed, and in example 2, the cow’s project is to go out of the pen and, perhaps, go to the milking robot. By “project” I mean what, according to my interpretation, the participants are trying to accomplish in this and other episodes. This attempt, their project, is longer than just one turn or one adjacency pair in the interaction. In example 1, the human participant is attempting to get the cow moving, and the cow is attempting to stay unmoving. These episodes are examples in which the projects of human and non-human animals conflict. The human participant, who is holding most, if not all, of the power in the cattle barn (see [53,59]), will eventually win.

### 4.2. Materiality of Language—Examples 1 and 2

**Figure 4:**
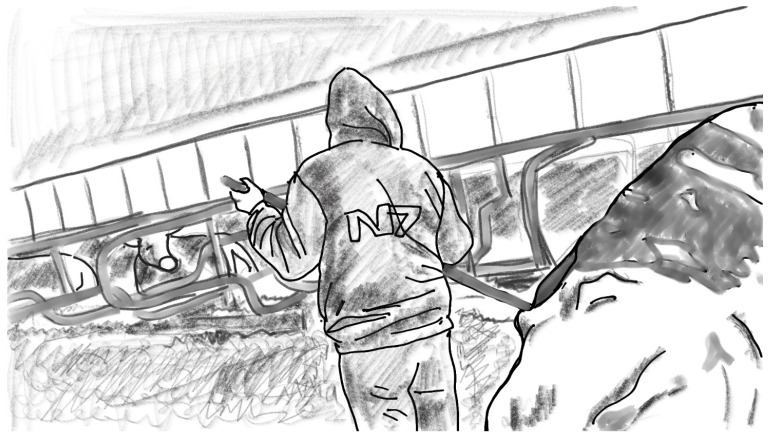
#1 Example 1.

Example 1 comes from Farm 1. In this episode, a farm worker, let us call them A, is cleaning the sleeping cubicles of cows. Because of this, the cows must stand up and move away. Before this episode, A has explained to me their working schedule, and cleaning the beddings is one of A’s duties in the morning. A tries to clean the bedding while the cows are away (cows may move freely in this farm), but at some point, it is necessary that A performs the cleaning. When this episode takes place, A is in the process of moving a whole row of resting cows. For this, A uses the dung pusher. In example 2 below, worker C pushed Marie (the cow) with the shaft of the pusher, but in this episode, A uses the edge of the pusher when tapping or touching the cow, Piipari. Some of the movements are not easy to see, but clearly some of the taps or beats on the side of the cow are harder, as they can be heard on the tape. In examples 1 and 2, touches and hits with the dung pusher are marked with the symbol “¿”. In accordance with multimodal transcription conventions [42,43], participants’ movements are marked with gray and the translation in italics. See Abbreviations in the very end of the paper for other markings (Figure 4).


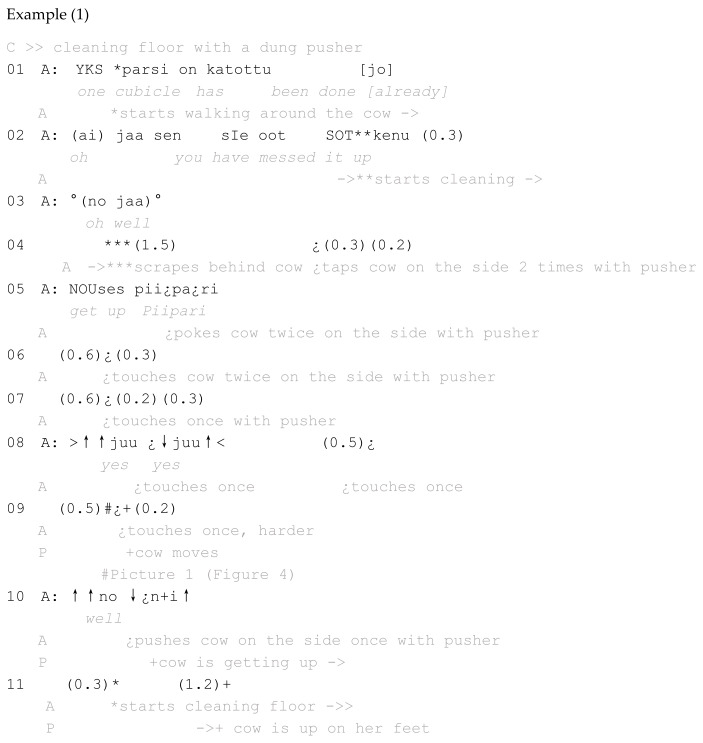


In example 1 we see many of the defining characteristics of talking to cows in the cattle barn. These include the use of short directives (*Nouses Piipari* and *juu juu*, lines 5 and 8), addressing a cow with the pronoun *you* (line 2), and using the cow’s name (line 5). (This worker knew all the 100 milking cows by their names as well as numbers and could tell them apart from a distance.) In line 2, A says, about Piipari’s cubicle, *(ai) jaa sen **sie** oot sotkenu*, ‘Oh, **you**’ve messed it up’. The pronoun *you* marks Piipari as an interlocutor and receiver of A’s words (also [3], p. 168).

Cleaning the cubicles is a familiar action to Piipari and the other cows on Farm 1, and they must know that they are to get up when the worker is cleaning their beddings. Yet this cow and many others are not quick to move. My interpretation is that, on one hand, the cows are not at all afraid of A, who is their principal stockperson and also strokes them, and on the other hand, standing up is, in fact, very difficult for dairy cows because of their heavy bodies and weak front legs, a byproduct of cows being bred to yield extensive milk quantities, and they do not want to stand up. Thus, successfully making a dairy cow move takes about 16 s in example 1.

A makes many attempts to make Piipari get up. First, in line 4, A taps the cow quickly twice with the dung pusher edge. A then says, using a directive in imperative, *Nouse-s Piipari* (rise.imp.2sg-ptcl Piipari), ‘Get up Piipari’, poking the cow twice on the side with the pusher. The cow does not move herself yet, and A gives her more nudges with the pusher, adding *juu juu*, ‘yes yes’ (line 8), which in this context is at the same time a directive and an affirmation that A really means that she needs to get up, affirming A’s previous turn as well as their bodily gestures (lines 5–7). However, A uses a higher and gentler voice in this turn. Most of the stockpersons in my data talk to cows, at least occasionally, with a special voice quality, which could be called ‘a cow voice’: it is a voice quality resembling baby-talk or pet-directed talk (see [4,60]). As Piipari still will not budge, A gives her three more taps, the last one being the hardest, after which Piipari shows signs that she will get up. A adds a concluding turn, *no ni*, ‘there’, pushing the cow once more with the pusher. (*No ni*, consists of two particles, *no* and *niin* in written Finnish, and is difficult to translate, as it is highly context-sensitive and could have dozens of meanings, depending on the action and position in a sequence or turn and prosody. In this example, it perhaps marks the action being almost complete, as the cow is just starting to move, and it could mean something like ‘That’s it’. In example 2 in line 3, *no ni* is in a different place, and its meaning is probably affirmative and positively evaluative; in line 13 another *no ni* is again concluding. (See [57], § 859.) After 1.5 s Piipari is fully up, which is not slow at all for a dairy cow.

**Figure 5:**
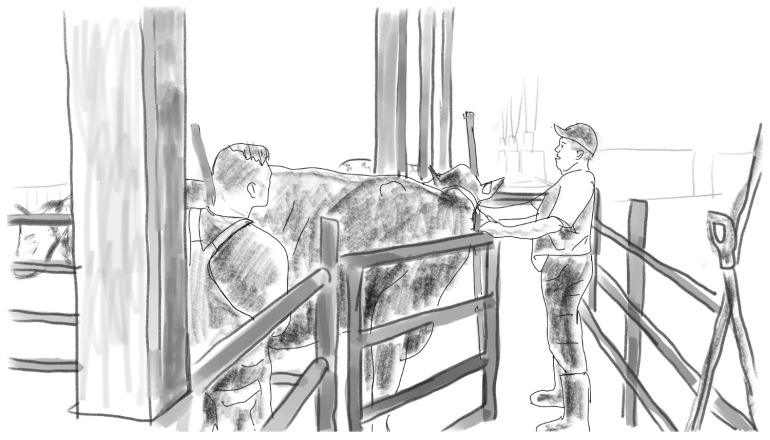
#1 Example 2.

In example 2, which comes from Relief workers, a worker, B, has taken a cow, Marie, back to the cattle barn from the pasture. Marie had given birth to a calf the previous night, but the calf had died. Marie has been taken to a pen, possibly for treatment. In this episode, B and another person, C, discuss the actions of Marie and what they mean. The dung pusher is used in its ‘shepherd’s-crook quality’ (see Section 5) (Figure 5).


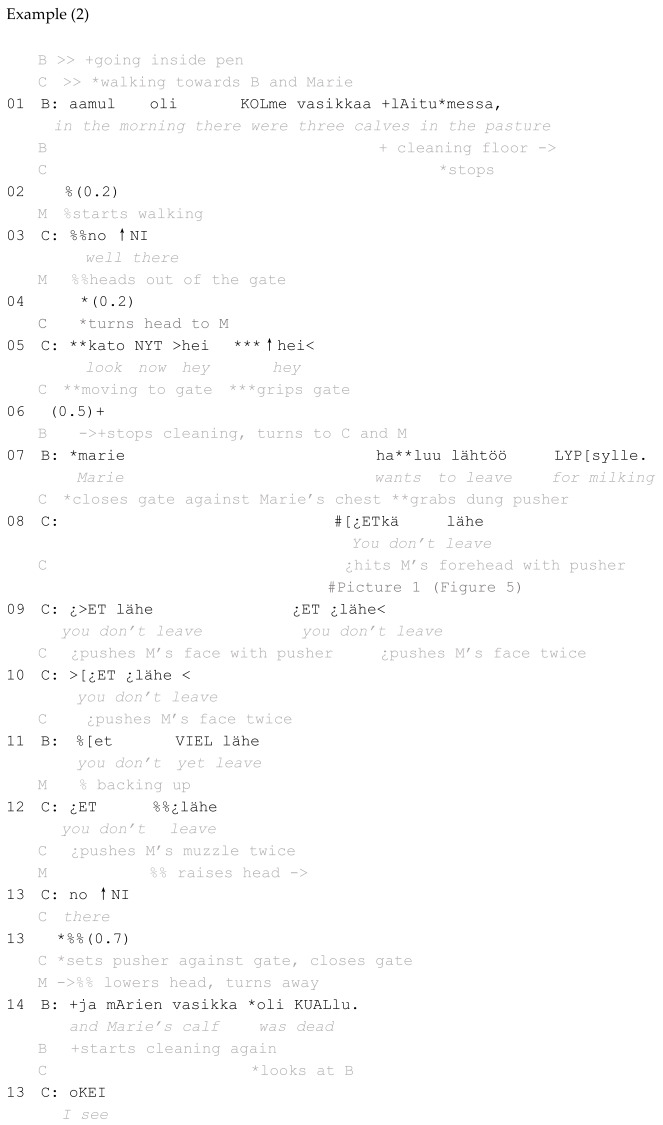


Example 2 is intriguing because it shows how speaker C uses both the linguistic and material resources in this context as affordances. An affordance is a resource that a subject uses for a certain purpose, something that they find meaningful and can manipulate in their Umwelt, in other words, “patterns for meaningful perception and action relative to the nature of the organism.” [1], (p. 626); (see also [45], pp. 4–5).

In this example, C notices that the cow, Marie, is trying to go out of the pen (line 4), as the gate has been left open by B, now cleaning the floor with a dung pusher. Immediately, C grabs another dung pusher kept nearby (line 7), and almost at the same time as C performs this bodily action, C also “grabs” a linguistic element from the turn of B (line 7), using it in again (line 8); thus, C uses the props and the linguistic context the same way, as affordances. They use the verb ‘to leave’ and the dung pusher for exactly the same reason, because they are already there.

In conversation analysis, linguistic material taken to use from a previous turn is sometimes called recycled (for example, [61], pp. 167–169, [62,63]). C’s use of B’s turn is this kind of lexical recycling. In line 7, B explains the reason for the cow’s behaviour: *“Marie haluu **lähtöö** lypsylle”,* ‘Marie wants to **leave** for milking’. (In dairy farms, according to producers and stockpersons, cows usually want to go to the milking robot or parlour for two reasons. Firstly, their udders become heavy and achy, partly because of the breeding that has focused on extensive milk production, and secondly, because they receive treats when they enter the milking robot or parlour.) C recycles B’s verb, apparently accepting their explanation, and says to Marie, pushing her with the dung pusher: *“Etkä **lähe** et lähe et lähe et lähe et lähe”*, repeating the command ‘You don’t **leave**’ (lines 8–12). While saying this, C pushes Marie rhythmically with the dung pusher’s shaft. In example 2, I have marked with “¿” the pushes or taps with the dung pusher performed by C (in lines 8–12).

B, then, again uses the verb *lähteä*, ‘to leave’, softening the directive used by C, perhaps because B feels sympathy towards Marie: *“Et **viel** lähe”*, ‘You won’t leave **just yet**’ (line 11). In this episode we see how the dung pusher is used as an affordance, but we see, too, how B is trying to understand the motivation of Marie’s actions and explain them. Perhaps B is using humour to lighten the situation, as B also uses a dialectal form of the verb *lähteä* (*lähtöö*, instead of *lähtee*), which could be seen as a humorous effort; B does not normally use this form. Another interpretation for this idiosyncratic dialectal form is that it marks the turn on line 7 as Marie’s voice; B, in fact, does quite a lot of ventriloquizing, pretending to speak as a cow (see [12]). Also, C’s following turn *Etkä lähe* (‘You don’t definitely leave’; ‘oh no you won’t!’) with its emphatic clitic particle -*kA*, could be interpreted as an answer to this imagined Marie’s voice. (See [64].) B seems to understand the action of C as well, as they evaluate what C has just said by repeating it partially, although changing it to be more favourable to Marie.

What about Marie herself? We do not really know what her motivation is, but it might just as well be the case that she is not trying to go to the milking robot, but just to move away, because she starts moving when B starts cleaning the floor (line 2). In some farms I visited, the workers moved cows away when they were cleaning the floor, as in example 1, often with the dung pusher, which B is holding too. Perhaps Marie is anticipating being pushed aside. When C has pushed her back into the pen, she raises her head, showing taking offence, not enjoying being pushed (line 12). Whatever she was attempting, she accepts the situation and turns away. However, there might be more interaction on the cow’s part than what can be seen in examples 1 and 2. One limitation with the research methods of this study is that the recorded footage does not always clearly show the cows’ expressions and small movements or reproduce very quiet sounds because of its quality and camera angles (see [47] on the importance of cows’ movements and sounds). Thus, this analysis is centred around the turns of the human participant, which are more clearly seen and heard. (This is a weakness that future research can hopefully ameliorate.)

### 4.3. Rhythm and Gesture-like Qualities in Dung Pusher and Snow Stake Use

Many episodes in my data, including examples 1 and 2 above, show that the use of the dung pusher is rhythmical: it expresses certain rhythms in accordance with either the bodily movements of the speaker or the linguistic units of spoken turns. People holding and using these sticks set the rhythm for their action and often give emphasis to what they are saying with the sticks.

Thus, in many episodes in my data, moving the dung pusher or snow stake shows a variation in beats. According to gesture studies, *beats* are a type of gesture that are rhythmically tied with elements of speech and often index a word or phrase they co-occur with as significant; beats give these elements emphasis. Beats, unlike many other gestures, have just two movement phases: up and down [32] (pp. 15–16, 81). Usually, beats are just a simple flick of the hand or fingers [32] (p. 15), but if a person is holding something in their hands, beats might be performed with these objects. In my data, a person might walk with a stick in their hand, talking, and at the same time, perform beats with it while talking.

In the episodes in which people use sticks when communicating with cows, their movements with the sticks are often rhythmically aligned with their speech. In example 2, C pushes Marie so that most of the pushes fall with the primary stress of the sentence (line 8, *ET-kä lähe*, no.2sg-ptcl leave, ‘you don’t definitely leave’); thus, their speech and gestures express the same rhythm. In example 1, one of the taps on the cow falls in rhythm with the second particle *juu*, which is, iconically, delivered in falling prosody (line 8 in example 1).

On the other hand, it is also typical that the material part of the action (the use of the pusher) is aligned so that it falls on the silent parts of the action, and not on speech turns, and this can be seen in example 1: when A speaks, they do not push, and most of the pushes are performed during speech pauses (see example 1 above, lines 4–9). The action or project of attempting to get the cow up remains the same during the silent and speech turn parts (see [65]). Gesture researcher David McNeill [32,66,67] has given the name *growth-point* to the specific idea that a speech element and a co-occurring gesture together present. A growth-point is something that they both, gesture and words, attempt to express. Definitely, when these movements performed with sticks co-occur with speech elements in these actions, they are born from the same growth-point, and essentially, they perform the same task. The actions and movements performed with sticks in these episodes are not what gesture studies mean by gestures (e.g., [32,66]), but they are similar to gestures in the sense that they share the same purpose as the speech elements. The same command, ‘get up’ (example 1) or ‘don’t leave’ (example 2), is performed with these two modalities, words and taps with the pusher (See [28]).

While talking about gesture-related qualities of dung pusher use, there is yet another way of using the dung pusher and the snow stake that is gesture-like: pointing. Pointing is one of the main types of gesturing, and it can be performed with a finger, hand, head, whole body, or an object (see [68,69]). Goodwin [69] (p. 219) writes, “A central locus for the act of pointing is a situation that contains at least two participants, one of whom is attempting to establish a particular space as a shared focus for the organization of cognition and action.” This shared space can be a section of the actual, physical space, as in the following examples, or it can be an abstract space, a concept that an interlocutor points at in order to single it out in conversation [32], (p. 18); [70]. I present two examples from my data in which an interlocutor points with a stick.

**Figure 6:**
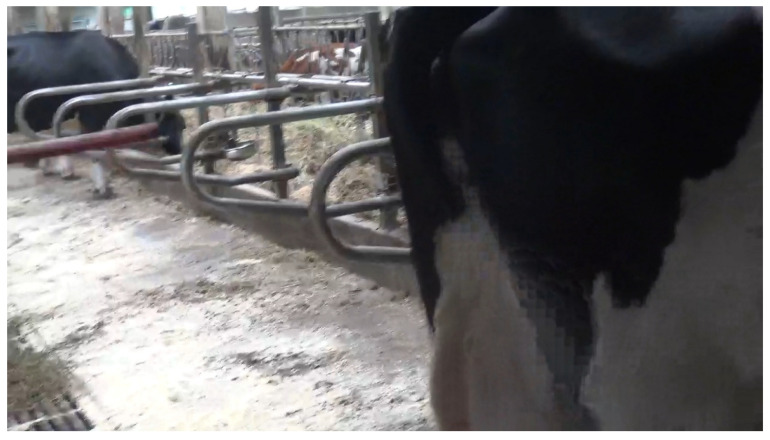
Example 3: Pointing with a snow stake. The orange snow stake can be seen on the left.

Example 3 comes from Farm 4. In this example, one of the workers points my attention to a blackboard above a cow’s cubicle. On this farm, each cow had their own place, and the duty of the workers was to see that the cows went to their own places when they walked into the barn from the pasture. For steering the cows to their own places, farm workers used snow stakes, and they each held a stick in their hand. This worker wanted to point my attention to the amusing name of a specific cow; she was named after a car type. They pointed with the snow stake to the blackboard where her name was written. Following their pointing, I instinctively turned my head and the camera to the pointed direction, thus only capturing one or two frames of the swift stick movement, seen in Figure 6.

**Figure 7:**
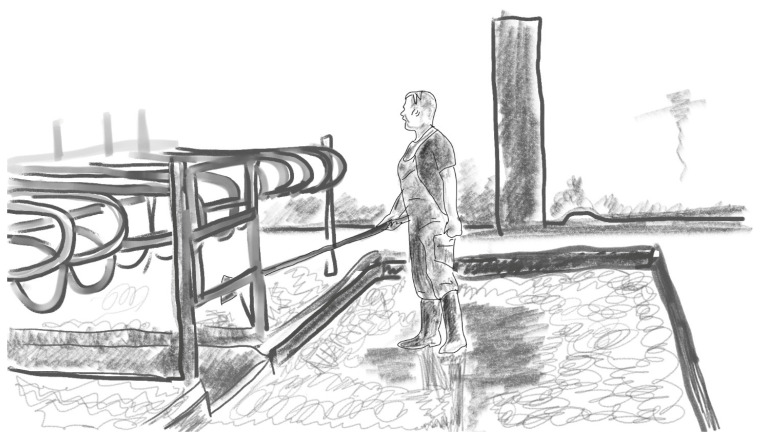
Example 4: Pointing with pusher.

In example 4, a farm relief worker (Relief workers) explains that cows are habitual creatures and always choose the same place, if possible. They point out with the dung pusher edge which cubicle a cow, Omena, will take when she comes. They then tap the floor twice, the first tap landing with the first syllable of the last word (see Figure 7), emphasizing the word *tähä*, ‘here’:

Example (4) “Omena kel on toi imunestolaite nenässä nih (.) mä päästän kohta ni se menee nukkuu **TÄhä**” ’Omena who is wearing that sucking preventor so (.) when I soon let her in so she well go sleep **here**.’

In example 4, the tap with a material object, the dung pusher’s edge, ties the action to a specific place in the material context, a specific cubicle, and more specifically, its floor as a cow’s resting place.

Thus, pointing with sticks and using them rhythmically and when emphasizing something is yet another example of the materiality of language use in the cattle barn. These objects—dung pushers, snow stakes—are affordances that are used, as well as tools, in many communicative ways.

## 5. Dung Pushers and Snow Stakes as ‘Meaning-Carriers’

In this section, I describe what meanings these tools, the dung pusher and the snow stake, acquire in the cattle barn. When explaining the uses of these tools, it is helpful to go back to von Uexküll’s [9] writing. Von Uexküll’s theory of meaning is helpful when we try to find a notion of meaning that could tackle the differing meanings that are available to differing living creatures, not just linguistic or symbolic meaning specific to human animals. Another starting point could be Peirce’s theory of signs, which, for example, Kohn [10] uses. For this examination, however, I find von Uexküll’s theory of meaning even more fitting because of its materiality and utmost generality. Some of Peirce’s ideas, such as the indexicality of signs, do come up in this study.

In his now-classic example, von Uexküll [9] (pp. 27–28) explains how a neutral object becomes a ‘meaning-carrier’, taking a paving stone as an example. For a walker on a country road, a stone is just a hard material that gives support to their feet. It has what von Uexküll calls ‘path-quality’. But if the stone is picked up and used in order to chase off an angry dog, it acquires quite a different meaning; when it is thrown at an angry dog, it acquires ‘throw-quality’. Von Uexküll [9] (p. 27) writes that “The stone lies in the objective observer’s hand as a neutral object, but it is transformed into a meaning-carrier as soon as it enters into a relationship with a subject.” When an object is used for something, it acquires meaning because of its function. It acquires meaning for the user, but in some cases, for other participants in the action as well, in these cases, the cow.

In my data, the dung pusher acquires the following qualities as a meaning-carrier for the human subject:

Floor-cleaner quality: A dung pusher is a multi-faceted meaning-carrier in the cattle barn. First of all, it of course has a ‘floor-cleaner quality’. The dung pusher is used for cleaning the floors, and that is why it is there in the first place. If one wants to buy a dung pusher, it can be found in the section for floor-cleaning tools in a hardware store.

Shepherd’s-crook quality: Sometimes the dung pusher, as a meaning-carrier, acquires a ‘shepherd’s-crook quality’. In most of the places in my data, the dung pusher was also used in order to steer moving cows. The dung pusher is a tool already in the hands of the workers, so why not use it in a way that is quite natural for a long stick: steering someone? Also, the dung pusher was often used when the farm workers wanted the cows to get up. First, they might say something, like “get up”, then touch the cow’s side with their rubber boots or hands, and finally, if the cow did not get up, they would touch the cow on the back with the stick or move the stick before her face.

Pointer quality: In some examples, the dung pusher is used as a pointer when a farm worker is pointing at something, in order to show it to me or someone else present. In these cases, the dung pusher acquires a ‘pointer quality’. Oblong objects that are already in one’s hand make excellent pointers.

Weapon quality: Perhaps one should not be surprised that the dung pusher can also acquire a ‘weapon quality’. ‘Weapon quality’ comes in two different nuances: as ‘self-defence-weapon quality’ and as ‘offensive-weapon quality’.

In my data, I did not directly see the dung pushers used as weapons. It would have been surprising if I had seen any kind of deliberate violence (apart from the structural violence that dairy farming is [53,59]). This is because I was invited to come to farms, and their participation was completely voluntary. Farms that had any issues in animal welfare would not have invited me in the first place. In a couple of episodes in my data, a worker uses a dung pusher in a way that could be gentler, to my unaccustomed eyes, as in example 2 in this study, but I would not call it beating. (Example 2 is a borderline use of the ‘weapon-quality’, however.) But some farm workers told me that they could be used in self-defence:

Example (5) “Sehän on sit semmonen, et sitä pitää välil niinku (…) Sen takiaha miul on aina paskakola, koska jotku lehmät o, siis vaikkei ne ole pahoja, mut sit jos ne tulee tiiet sie oikei niinku, et ne siis tuuppaa päällä, se sattuu aivan mielettömästi ni, mieluummin on sit joku millä niitä vähän niinku.”

‘The case is, that one needs to occasionally, like (…) That is why I always have a shit pusher with me, because some cows are, even though they are not bad, but if they come, you know really like, that if they come pushing with their head, it hurts like crazy, so, I would rather have something with I could use like.’ (Farmworker, Farm 1.)

Using sticks for self-defence is also acknowledged in research [20] and a research-based guidebook about safety in animal farming: ’If a cow attacks toward a worker, they should have something in their hands for self-defence. For example, a stick, broomstick or dung pusher is appropriate for this use’ (“Jos nauta hyökkää kohti hoitajaa, hoitajalla tulisi olla jotain kädessään itsepuolustusta varten. Esimerkiksi keppi, harjan-varsi tai lantakola soveltuvat käyttötarkoitukseen”.) [71], (p. 10).

Interestingly, *speaking* has this same quality of a method of self-defence in certain cases. Speaking could function proactively as an alarm for the cows that the worker is approaching, thus helping in the worker’s self-defence. If the cows do not know that the worker is approaching, they might be frightened and kick. Many workers whom I interviewed had had injuries and even broken bones because of cows kicking or pushing (see [20]). One worker (Farm 2) told me the following:

Example (6) “Mä puhun lehmille enemmän kun mun työkaverit koska (.) mä en haluu enää yhtään potkua, mulla on nivelrikko ja särkee nivelet ilman potkujaki.”’I talk to cows more than my colleagues because (.) I don’t want any more kicks, I have osteoarthritis, and my joints hurt without the kicks, anyway.’ (Farm worker, Farm 2.)

However, dung pushers are used when beating the cows as well, and the tool then acquires an ‘offensive-weapon quality’. There are accounts according to which cows are hit with dung pushers on a routine basis, and even students learning to take care of cows are taught where to hit them: “Sanottiin kyllä, että sellaisella paskakolalla kun hakkaa jalkoihin, niin kyllä ne lehmät parrelta nousee”, ‘They said that when one beats them on the legs with that kind of shit pusher, then the cows get up from their bed’ [23], (pp. 112–113; quote p. 112.). The workers who beat cows with dung pushers are probably aware of its ‘weapon-quality’ very well. Perhaps, for them, the tool is there especially for that purpose.

What about the snow stake? What are its meaning qualities?

**Figure 8:**
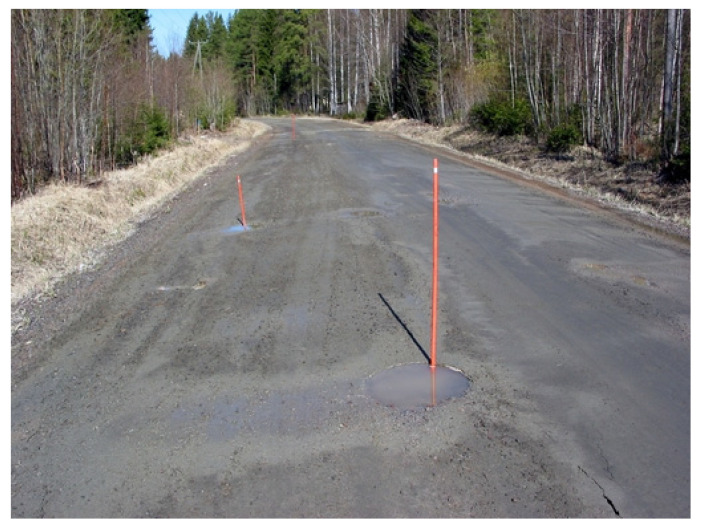
Snow stakes marking potholes. (TH Picture 10223, by Anja Laine, CC BY-NC-ND 4.0) Picture obtained through finna.fi, with direct address https://www.finna.fi/Record/mobilia.mobilia.collectionpro.fi:TH_Valokuva%2FTiehallinto%20kuvat__10223?sid=5167627001&lng=en-gb (accessed on 1 September 2025).

Marker quality: Obviously, the raison d’être of the snow stake is to mark the road edges in the winter. It is used to mark other objects that should be noticed too, such as potholes in roads throughout the year, as can be seen in Figure 8. Whatever it marks, as a meaning-carrier, it acquires the ‘marker quality’.

Shepherd’s-crook quality: The snow stake also has a ‘shepherd’s-crook quality’, as explained in Section 4.1. Using snow stakes when steering cows was conventional. Using dung pushers for steering animals is almost self-evident, because the workers are already using them for floor cleaning. Using snow stakes for cow steering means that this use has to be planned, and the workers keep the sticks there expressly because of their ‘shepherds-crook quality’.

Pointer quality: Additionally, when it is needed, the snow pole has, as we saw in Section 4.3, the ‘pointer quality’.

Walking-stick quality. In addition, a snow stake is the right height for a walking stick. Thus, it also has a ‘walking-stick quality’, as workers lean on the snow stakes (and other sticks of the same height) that they are holding when working with cows. This, too, can be seen in my data.

**Figure 9:**
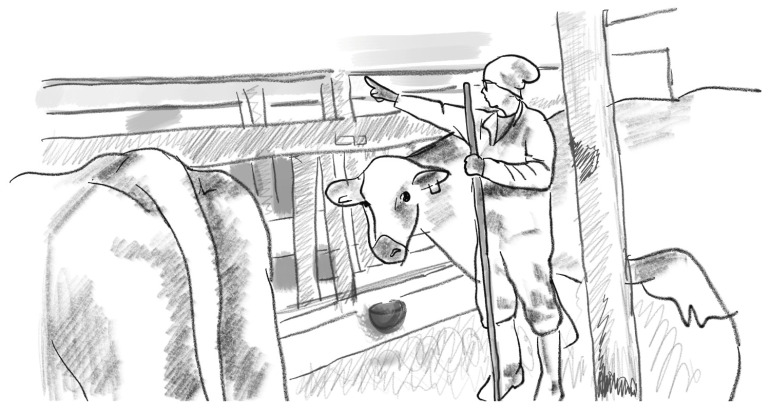
Pointing with hands, Farm 2.

Different qualities of the sticks do not show everywhere. For example, on Farm 2, the farm worker, as far as I could tell, only used the dung pusher in order to push dung into and along the gutters, and thus the ‘floor-cleaner quality’ was the only quality that the dung pusher acquired. On Farm 2, there was no need to steer the cows, as they were attached to their stalls, and the worker used their hands and body when pushing the cows when operating the milker. Thus, they did not need the ‘shepherd’s-crook quality’ of the dung pusher. In addition, this farm worker only used their hands when pointing (see Figure 9). This could be because the cattle barn was small and narrow, and the pusher was relatively long and heavy; there was no space to lift it up. Thus, again, the material surroundings of actions are significant.

At the beginning of my observation, the use of the dung pusher seemed, as it were, opportunistic to me; it seemed to me that it was used because it was there. This is not the entire truth, however. The workers were familiar with the ‘shepherd’s-crook quality’ of the pusher, and they were aware of its quality as a potential self-defence weapon. These qualities were mentioned in the literature. (For example, [20,23].) In addition, the use of snow stakes made it evident that these sticks were there especially for the purpose of steering cows. When cows were farmed in a more traditional way and were pastured in natural meadows and forests, their herders would use sticks and twigs of different kinds when moving their animals and working with them [4]. The practice is age-old, but modern-day farming has changed what they have at hand now: dung pushers and snow stakes.

The final question, though, is whether the dung pusher is a meaning-carrier for cows? That depends on the meaning a dung pusher might acquire for an individual cow during her life. For a cow regularly beaten with a dung pusher, it probably is a meaning-carrier. On Farm 1, the cows were familiar with dung pushers but did not seem to be bothered by them, as we saw in example 1. It is hard to tell whether the tool is a meaning-carrier for them. On Farm 2, the dung pusher was probably not a meaning-carrier, as the cows had little contact with it. However, on Farm 3, the dung pusher might have been a meaning-carrier for the cows as well.

On Farm 3, cows are moved in groups of 50 cows into a separate milking parlour located in another hall, through a corridor, three times a day. A cow or a group of cows can be moved by using their flight zone and balance point. Cows can show avoidance or approach behaviours for many reasons, their temperament, size of the herd, and prior experiences each playing a role. However, the main influence for a cow’s avoidance behaviour is often the lack of contact with humans or the aversive quality of that contact: if a cow receives plenty of pleasant contact with humans, they are less avoidant [72]. Thus, if a cow is familiar with her stockpersons and has good prior experiences (as in Farm 1), her flight zone is minimal, but if a cow is unfamiliar with a stockperson or people in general or has bad experiences with them, her flight zone can be several meters, and a cow will move away from an approaching human. [73] In addition to the flight zone, cows can be moved by taking their point of balance into consideration. The point of balance is an imaginary line at the animal’s shoulders. Walking behind a cow’s point of balance keeps a cow moving, but walking in front of their point of balance makes a cow turn [71], (pp. 13–14) [73]; Farm 3 used these instincts when moving cows. Furthermore, the owner of Farm 3 had specifically told the workers *not* to talk to cows when the cows were moved to the milking parlour or while milking. In the opinion of the owner, talking to cows would interfere with the task, as this farm wanted to use cows’ flight zone and keep their avoidance behaviour. According to the owner of Farm 3, by using their flight zone, the cows could be moved as a herd more easily, and if cows were tamer and more reliant on humans, they would not move as readily when a farm worker approached them. (I will come back to this instruction in Section 6.)

**Figure 10:**
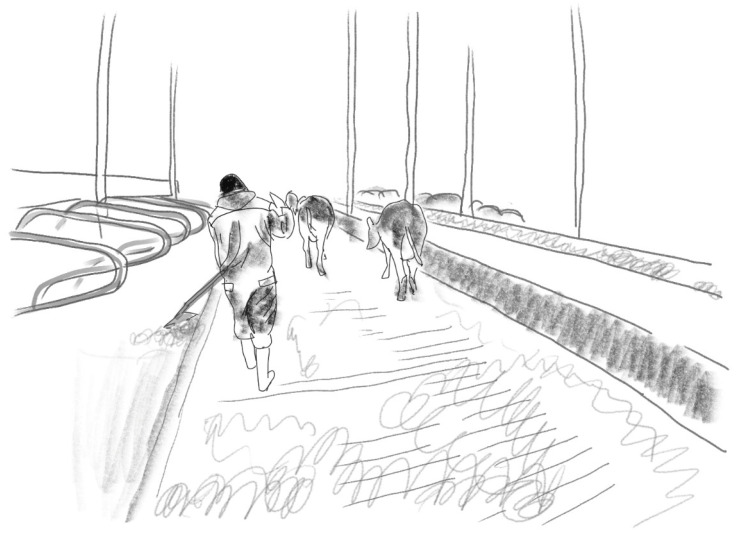
Moving cows on Farm 3.

Also, a cow becomes startled easily if approached from behind, or faced head-on, when they are not accustomed to humans [71], (pp. 14–15); Ref. [73]. Cattle do not appreciate objects hovering somewhere at the edge of their visual field. Thus, sticks are practical when moving cows, because one does not need to touch the cow; it is enough that one waves a stick near them or their head [71], (p. 15). There is one episode from Farm 3 in which the worker touches the face of a cow with the dung pusher edge, thus making her move. In the footage from this farm, the stockperson has a double job: to clean the floor and beddings and to make the cows move. Approaching the cows with a pusher in their hand was mostly enough to get the cows moving. See Figure 10.

On this farm, the owner recognized *the sound of the dung pusher scraping the floor* as a meaning-carrier for cows. The owner explains, as the other stockperson is moving the cows and using the dung pusher’s ‘shepherd’s-crook quality’,

Example (7) “Se tämä varsinainen kerääjä ei oo sanonu sanaakaa eikä oo viheltäny eikä tehny yhtääm mitääm muuta ku tuo (.) kola’ ’ääni on ainoo mikä tuntuu niitä liikuttavan (.) ku kolan kola’ ’ääni lähestyy nii sitte ne liikkuu.” ’This actual collector [of cows] has not said a word and has not whistled or done anything else than that (.) sound of the pusher is the only thing that seems to move them (.) when the sound of the pusher approaches then they move.’ (Owner, Farm 3.)

Thus, this cattle barn owner recognized the dung pusher or, more precisely, its sound as a meaning-carrier for their cows; it was “the only thing that seems to move them”. This is not a stand-alone example in which a farm worker tries to understand what motivates cows and what they are thinking; in my research data, there are many instances in which workers orient themselves to the subjective meaning universes of cows. For example, this owner also recognized the colour of the jacket of the farm workers as a meaning-carrier for cows; the owner was wearing the wrong-coloured jacket, thus surprising the cows.

## 6. Different Methods of Communication

As a linguist, I am interested in language and the linguistic activity that the animals I study take part in. However, as we have seen, much of the communication on farms is not linguistic per se, as it revolves around other kinds of meanings and signs, such as routines (e.g., milking and feeding, with their separate phases), touches, sounds, and very likely smells. Therefore, choosing lexical communication is not the only option for a stockperson. Farm 4 showed an example of a place where most of the activity happened without words. For example, most of the milking in Farm 4 relied on only touching the cows without using spoken words. The activity on this farm was based on routines that were always performed in the same way, with certain phases, such as touching the cow when she needs to get up and wiping the udders in a certain way, and so on. It was not entirely silent, however, as workers did use clattering and taps against metal and other auditory signals, for example. As already mentioned, out of eight episodes of steering cows with snow stakes on Farm 4, only one included words.

On Farm 3 workers were forbidden to *talk* to cows while they were moved and milked. The owner thought that it was best to regard his cattle as herd animals and let them keep their natural instincts; they found that talking to animals was not natural in this sense. However, the owner also told me that, when forbidden to talk to the cows, the stockpersons had started to *whistle* to the cows as a signal when opening the gate to the corridor leading to the milking parlour. The owner had allowed them to whistle.

To me, this was telling. Whistling and talking are basically the same actions in this context. Whistling is an index and not unlike the spoken directives found in my data. The only difference is that it is not composed of phonemes. In fact, whistles and some other non-lexical “sound objects” are not self-evidently non-lexical, but are on a formal and functional continuum between non-lexical and lexical sounds [74], (pp. 167–167), and whistles can have a variety of interactional meanings [74]. In addition, directives directed at animals, especially dogs and horses, often include whistles [3,4,74]. The human species is essentially a talking species; humans engage in talk in almost all situations. Not talking in certain activities is difficult, and calling someone from a distance is one of those situations. Thus, the spoken directives, which are in fact also indexical, were substituted with indexical whistling.

## 7. Conclusions

In this paper, I have described how dung pushers, snow stakes, and other sticks are used on present-day Finnish dairy farms when workers wish to make cows move, and how these material means are used in combination with linguistic means. I have also discussed other-than-linguistic meanings and ways of communication that are available to humans and cows, such as routines and touches.

This study showed some instances in which people actively communicated with cows. In Example 1, stockperson A wanted to move a cow, but the cow did not want to move. A used many resources in their attempt to make the cow move, such as spoken directives, “cow voice”, and taps with a dung pusher. Certainly, there was an imbalance of power. But if we look at the timing of A’s spoken turns and their movements, we see that A also gives the cow, Piipari, time to react and take *her* turn. There is interaction.

I have been asked whether my study is about animals or about humans. To me, my research is about people orienting to the Umwelts of different species, of people trying to understand other living creatures, which I find valuable and worthwhile to study. This specific study was about one of our companion species, cattle, but in my ongoing research project data, people have tried to orient to the subjective meaning universes of quite remote species, such as insects, fish, and octopuses. People have tried to understand other minds and, if not quite able to do so, they are at least willing to give it a try. At the beginning of this article, I referred to Kusters et al. [27], who suggest that we could speak of semiotic repertoires when referring to the various multimodal (linguistic as well as material) resources individuals use when communicating, and Cornips [45] has suggested that the concept of semiotic repertoires can also be extended and applied to non-human animals when studying multi-species interactions and the agencies of non-human animals. However, to grasp the semiotic repertoires of a non-human animal would require that we had access to the senses and sensations of our non-human companions, and we are limited by our bodies, our brains, and our own Umwelts [9]. We can try to understand individuals of other species that we meet, to decipher their gaze, movements, sounds, expressions, gestures, and intentions, for example, but our efforts might remain limited (see [64,75]). Despite the limitations of our own senses and brains, trying to understand and empathize with non-human animals is an ethical attempt we should make in order to appreciate the life and biodiversity of this planet.

## Figures and Tables

**Table 1:** Conditions on Farms 1–4.

Condition 1. *Free-stall cattle barn, milking performed by robots*: Cows move freely in and out, and choose when they are milked. (Farm 1)	Condition 3. *Tie-stall cattle barn, cows’ season inside:* Cows are all in their separate stalls and not moving freely. (Farm 2)
Condition 2. *Free-stall cattle barn, milking performed at a milking parlour:* Cows move freely inside their barn, but are moved in groups of 50 cows into a different space to be milked. (Farm 3)	Condition 4. *Tie-stall barn, cows’ season outside:* Cows are out in the pasture and walk into the cattle barn to be milked and are attached to their cubicles while milked. (Farm 4)

## Data Availability

The data presented in this article are not available because of data protection constraints.

## Abbreviations

2SG	2nd person singular
IMP	imperative
PASS	passive
PTCL	particle
Transcription conventions for speech and movements. Adapted from [42,43].	
(.)	micro-pause
(1.5)	measured pause
LÄhe	primary pitch accent
lÄhe	secondary pitch accent
°no jaa°	quiet speech
>et lähe<	fast speech
↑/↓	rise/fall in pitch
[	overlap (start)
]	overlap (finish)
(ai)	doubt in transcription
	level pitch
.	falling pitch
>>	an action started before the transcribed excerpt
¿	hit or touch with a pusher
*/**/***/	embodied actions begin in relation to speech (interlocutor 1)
+/++/+++	embodied actions begin in relation to speech (interlocutor 2)
%/%%	embodied actions begin in relation to speech (interlocutor 3)
->	embodied action continues until
->>	an action continues beyond the transcribed excerpt
#	indicates the exact moment at which a screenshot was taken
